# Therapeutics of Gold

**Published:** 1892-11

**Authors:** 


### Therapeutics of Gold.

Dr. John Strahan in *Brit. Med. Jour.*, says: The best recent writers agree with the old authorities that gold will cure old cases of syphilis where repeated courses of mercury and potass. iodid. have failed. For instance, it is the best remedy in ulcerations of the throat, syphilitic ozena, diseases of bones, and syphilitic phthisis. At present gold is principally used for present neuroses, impotence, etc. Niemeyer used it much in hysteria, and Noggerath says it quickly cures chronic ovaritis, if uncomplicated. Gold salts, if pushed, produce salivation, which however, can always be distinguished from that due to mercury by not affecting the teeth, cheeks or gums. They seem to produce a more active cerebral circulation. At all events, the effect of gold upon the brain is remarkable. The intellect becomes much more active, great cheerfulness, or even mental excitement, like mild alcoholic intoxication, results. Gold salts are said to be aphrodisiac.

